# Identification of the ferroptosis-related ceRNA network related to prognosis and tumor immunity for gastric cancer

**DOI:** 10.18632/aging.204176

**Published:** 2022-07-14

**Authors:** Zhiping Xiang, Xingguo Zhou, Geofrey Mahiki Mranda, Ying Xue, Yu Wang, Tian Wei, Junjian Liu, Yinlu Ding

**Affiliations:** 1 Department of Gastrointestinal Surgery, The Second Hospital, Cheeloo College of Medicine, Shandong University, Jinan, Shandong, China

**Keywords:** competing endogenous RNA network, ferroptosis, gastric cancer, overall survival, immune infiltration

## Abstract

Gastric cancer (GC) is a highly invasive course and has a very poor prognosis. Because there are no obvious symptoms in the early stage, most patients with GC are diagnosed in the late stage. The effective diagnosis, prognosis biomarkers and treatment targets of GC can solve this problem to a great extent. Although researchers have done a lot of research on GC in recent years, the relationship between the competing endogenous RNA (ceRNA) network of ferroptosis-related genes and the GC remains to be explored. Therefore, the research done in this paper has become particularly important. Download the expression data and clinical survival data about stomach adenocarcinoma from UCSC Xena and The Cancer Genome Atlas (TCGA) platform. Using bioinformatics tools to screen lncRNAs, miRNAs and mRNAs that are differentially expressed in GC samples and normal samples and related to the prognosis of GC. Then, screening lncRNAs, miRNAs and mRNAs with targeted relationships from the Starbase database. Subsequently, correlation analysis and survival analysis were carried out respectively. Finally, we get a ceRNA network related to the prognosis of GC patients. Cell experiments confirmed the results obtained by bioinformatics. This is critical for the discovery of the diagnosis, prognosis biomarkers and treatment targets.

## INTRODUCTION

**Figure 1:**
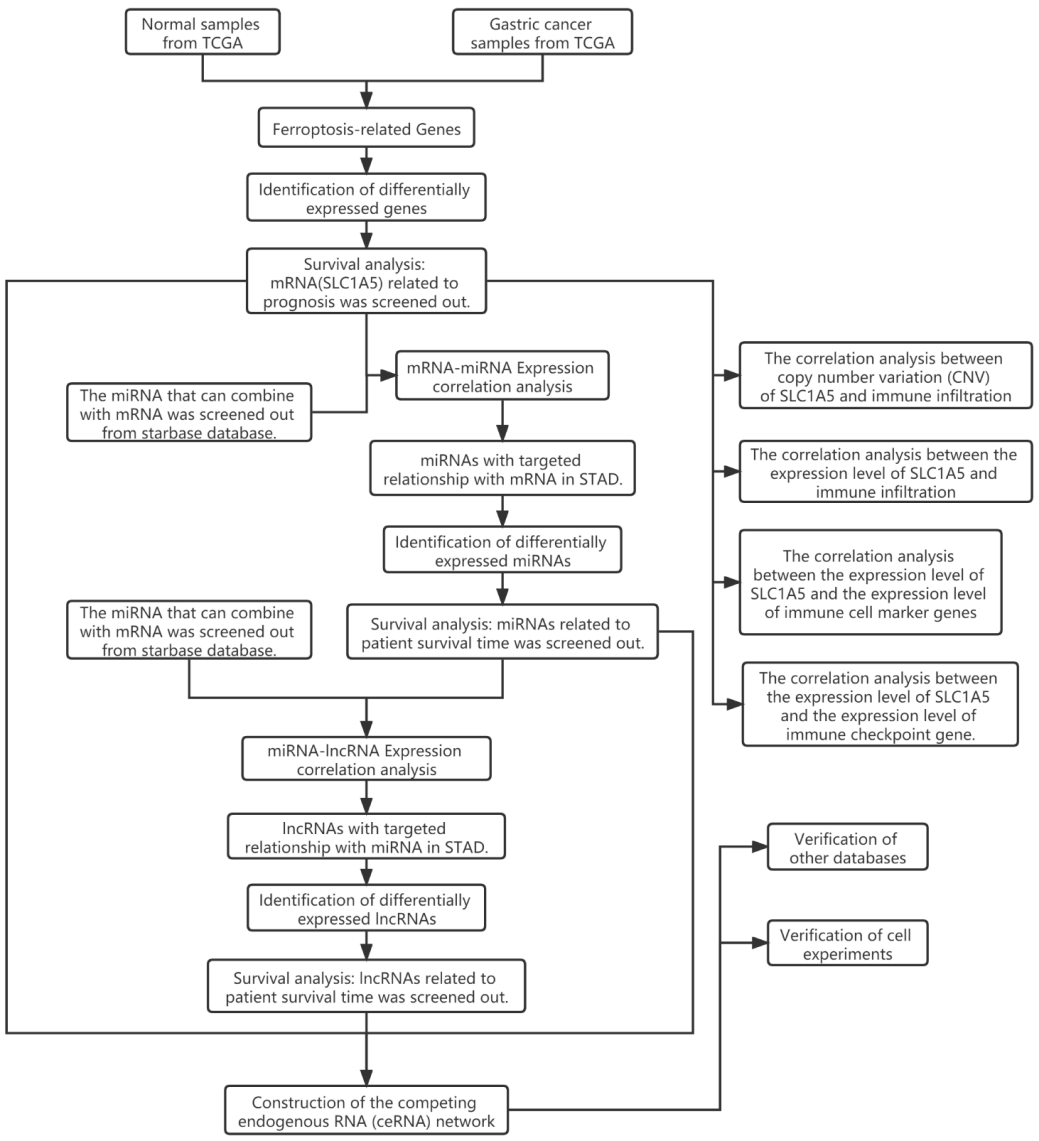
Flow chart of this research showing steps involved in the construction of competing endogenous RNAs networks.

According to the latest global cancer statistics, stomach cancer has more than one million new cases and an estimated 769,000 deaths worldwide in 2020, ranking fifth for incidence and fourth for mortality globally [1]. Because there are no obvious symptoms in the early stage, most patients with GC are diagnosed in the late stage. The effective biomarkers for the diagnosis and prognosis of GC can solve this problem to a great extent. The occurrence and progression of GC is a complex and multifaceted process, involving many molecules and factors. In recent years, researchers have made a deep exploration of the potential diagnosis, prognosis biomarkers and treatment targets of GC. The researchers observed that ferroptosis was associated with tumor prognosis [2]. Ferroptosis is a newly discovered form of regulating cell death, which is driven by lipid peroxidation and the accumulation of lethal reactive oxygen species [3]. Cancer cells usually have defects in the process of cell death, so ferroptosis-induced cancer cell death has gradually become a promising treatment [4]. In addition to the related studies on ferroptosis, the ceRNA hypothesis [5] proposed by Salmena et al. has also had a far-reaching impact. They believe that multiple RNAs can interact through microRNA (miRNA) response elements (MREs), and ceRNAs includes various types of RNAs, such as circRNAs, long non-coding RNAs (lncRNAs), miRNAs, and protein-encoding mRNAs. The ceRNA network combines the function of the protein-coding mRNAs with cyclic RNAs, miRNAs, lncRNAs and other non-coding RNAs [6]. When some RNAs in the ceRNA network are abnormally expressed, it can competitively affect the expression of other RNAs, resulting in possible interference of cell physiological functions, which may have a far-reaching impact on the occurrence and progression of GC. In tumors, ferroptosis-related genes and ceRNAs have important biological functions. Although researchers have done a lot of research on GC in recent years, there is still a lack of research on the ceRNA network of ferroptosis-related genes. The function and regulatory mechanism of ferroptosis-related genes and ceRNAs in GC have not been fully elucidated. The relationship between the ceRNA network of ferroptosis-related genes with the diagnosis, treatment and prognosis of patients with GC remains to be studied. Therefore, the research done in this paper has become particularly important. Bioinformatics is a subject that studies the laws of biological systems using mathematics, informatics, statistics and computer science. Biological data are collected, screened, processed and utilized by computers. The reliable results can be obtained by bioinformatics analysis of a large number of data in the public database. Cell experiments can verify the bioinformatics results and increase the reliability of the conclusions. The purpose of this study is to comprehensively analyze the expression of ferroptosis-related mRNAs, miRNAs and lncRNAs in GC by bioinformatics data, and to construct the mRNA-miRNA-lncRNA regulatory network of ferroptosis-related genes. This is critical for the discovery of the potential diagnosis, prognosis biomarkers and treatment targets of GC, and understanding of the functional and regulatory mechanisms of ceRNAs. The expression data and clinical survival data of GC were derived from UCSC Xena. There were 407 samples, including 32 normal samples and 375 GC samples. The clinical survival data included the survival time and survival status of patients with GC, with a total of 502 samples, including 88 normal samples and 414 GC samples. The miRNA data were collected from TCGA. There were 491 samples, including 45 normal samples and 446 GC samples. Through bioinformatics analysis and cell experiment verification, a simplified ceRNA network was constructed and the relationship between ferroptosis-related genes and ceRNAs was systematically analyzed. The ceRNA network was composed of 1 lncRNA, 1 miRNA and 1 ferroptosis-related mRNA. At the same time, the potential biological function of differentially expressed ferroptosis-related genes was identified, and it was found that SLC1A5 was closely related to tumor immunity. Through this analysis, we aim to better understand the potential diagnosis, prognosis biomarkers and treatment targets of GC. The flow chart of this study is shown in Figure 1.

## RESULTS

### The ferroptosis-related genes that are differentially expressed and related to prognosis

32 normal samples and 375 GC samples were compared. Data processing revealed that there were four ferroptosis-related genes (including FANCD2, NOX1, SLC1A5 and TFRC) with log_2_FC > 1, FDR<0.05 and four ferroptosis-related genes (including AKR1C2, AKR1C1, CRYAB and MT1G) with log_2_FC < -1, FDR < 0.05 in GC samples. Eight differentially expressed ferroptosis-related genes are shown in Supplementary Table 1. Survival analysis of eight differentially expressed ferroptosis-related genes was carried out. Through data processing, it is found that the ferroptosis-related gene (SLC1A5) was related to patient survival (p-value < 0.05, Supplementary Table 2). Finally, the genes SLC1A5 up-regulated in GC samples and related to prognosis were obtained.

### The miRNAs with the targeted relationship with mRNAs

**Figure 2:**
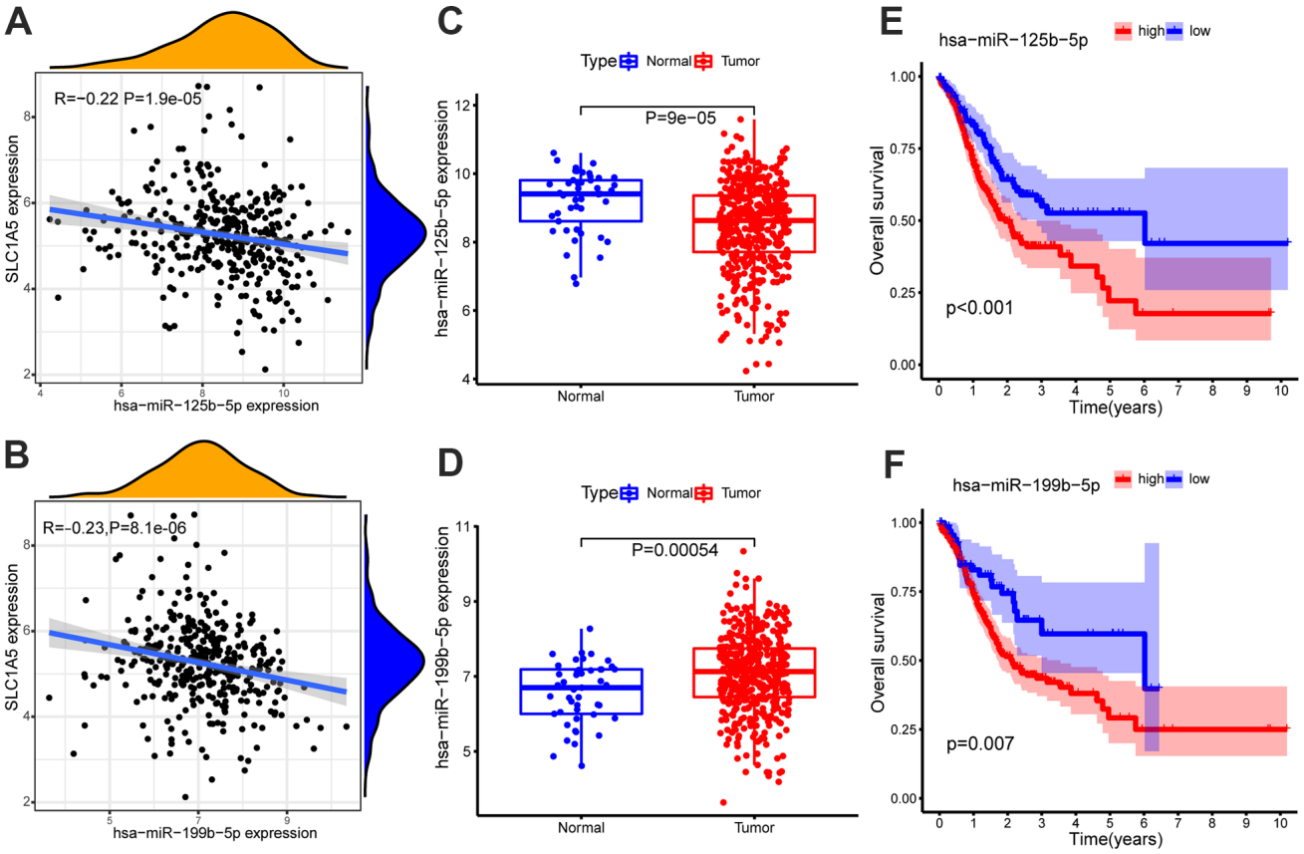
Expression correlation analysis of SLC1A5 and (**A**) hsa-miR-125b-5p, (**B**) hsa-miR-199b-5p. Differential expression analysis of (**C**) hsa-miR-125b-5p, (**D**) hsa-miR-199b-5p in normal samples and GC samples. The GC samples were divided into high and low expression groups according to the miRNA expression level. Survival analysis of (**E**) hsa-miR-125b-5p, (**F**) hsa-miR-199b-5p. Survival analysis shows that hsa-miR-125b-5p and hsa-miR-199b-5p are associated with overall survival (OS). The x-axis depicts the time of overall survival and the y-axis depicts the cumulative survival rate.

The miRNA dataset involves 491 samples, including 45 normal samples and 446 GC samples. The miRNA binding to SLC1A5 was screened from the Starbase database, and two miRNAs (including hsa-miR-125b-5p and hsa-miR-199b-5p) that were differentially expressed and negatively correlated with SLC1A5 were obtained by expression correlation analysis and differential expression analysis. Survival analysis showed that these two miRNAs were all related to the survival time of patients with GC (p-value < 0.05, Supplementary Table 3). In patients with GC, the low expression of hsa-miR-125b-5p and hsa-miR-199b-5p can relatively prolong the survival time. The correlation scatter diagram, differential expression box diagram and survival curve are shown in Figure 2. Among them, hsa-miR-125b-5p meet the screening criteria of expression correlation analysis and differential expression analysis (COR <-0.2, p-value < 0.01; log_2_FC < 0, FDR < 0.05, Supplementary Table 4).

### The lncRNAs with the targeted relationship with miRNAs

**Figure 3:**
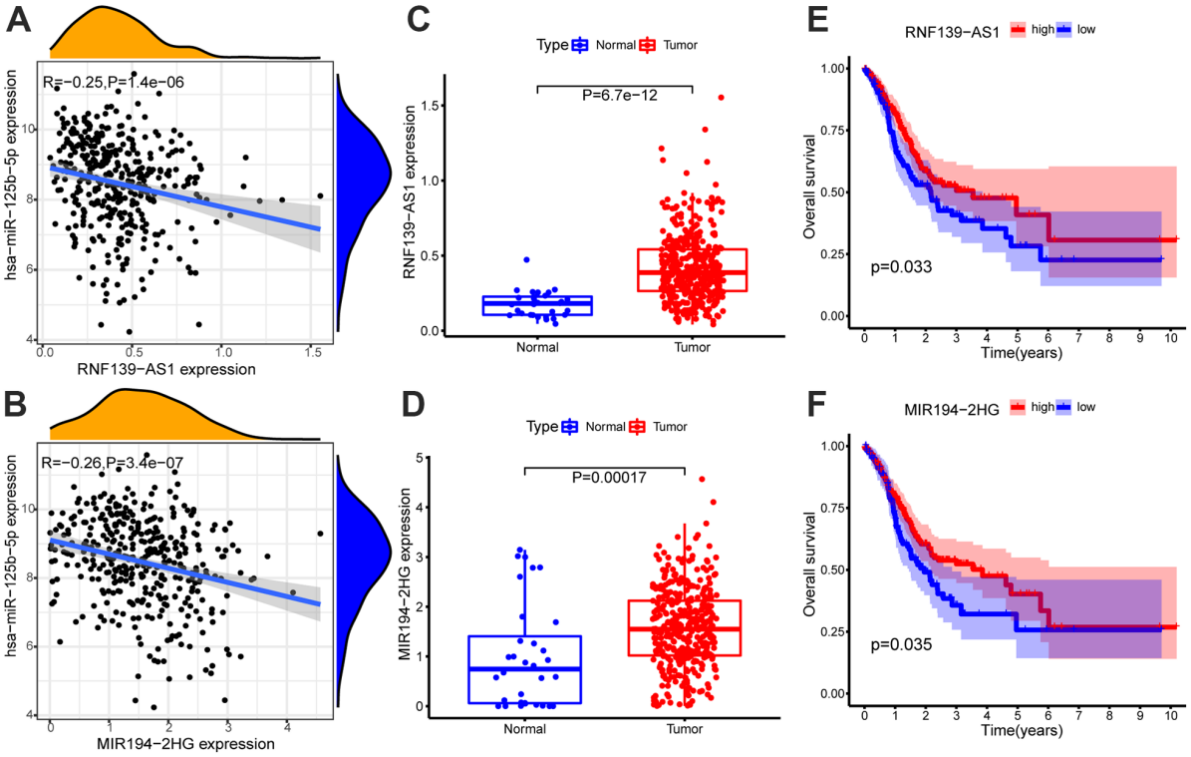
Expression correlation analysis of hsa-miR-125b-5p and (**A**) RNF139-AS1, (**B**) MIR194-2HG. Differential expression analysis of (**C**) RNF139-AS1 and (**D**) MIR194-2HG in normal samples and GC samples. The GC samples were divided into high and low expression groups according to the lncRNA expression level. Survival analysis of (**E**) RNF139-AS1, (**F**) MIR194-2HG. Survival analysis shows for RNF139-AS1 and MIR194-2HG are associated with overall survival (OS). The x-axis depicts the time of overall survival and the y-axis depicts the cumulative survival rate.

The lncRNAs combined with hsa-miR-125b-5p were screened from the Starbase database, and two lncRNAs (RNF139-AS1 and MIR194-2HG) that met the screening criteria (COR <-0.2, p-value < 0.01; log_2_FC > 0, FDR < 0.05) were obtained by expression correlation analysis and differential expression analysis (Supplementary Table 4). Survival analysis showed that these two lncRNAs were related to the survival time of patients with GC (p-value < 0.05, Supplementary Table 3). The expression of RNF139-AS1 and MIR194-2HG were relatively up-regulated in GC samples. In GC, the survival time of GC patients with low expression of RNF139-AS1 and MIR194-2HG was relatively shorter than that of GC patients with high expression. The correlation scatter diagram, differential expression box diagram and survival curve are shown in Figure 3.

### Experimental verification of the results of bioinformatics analysis

**Figure 4:**
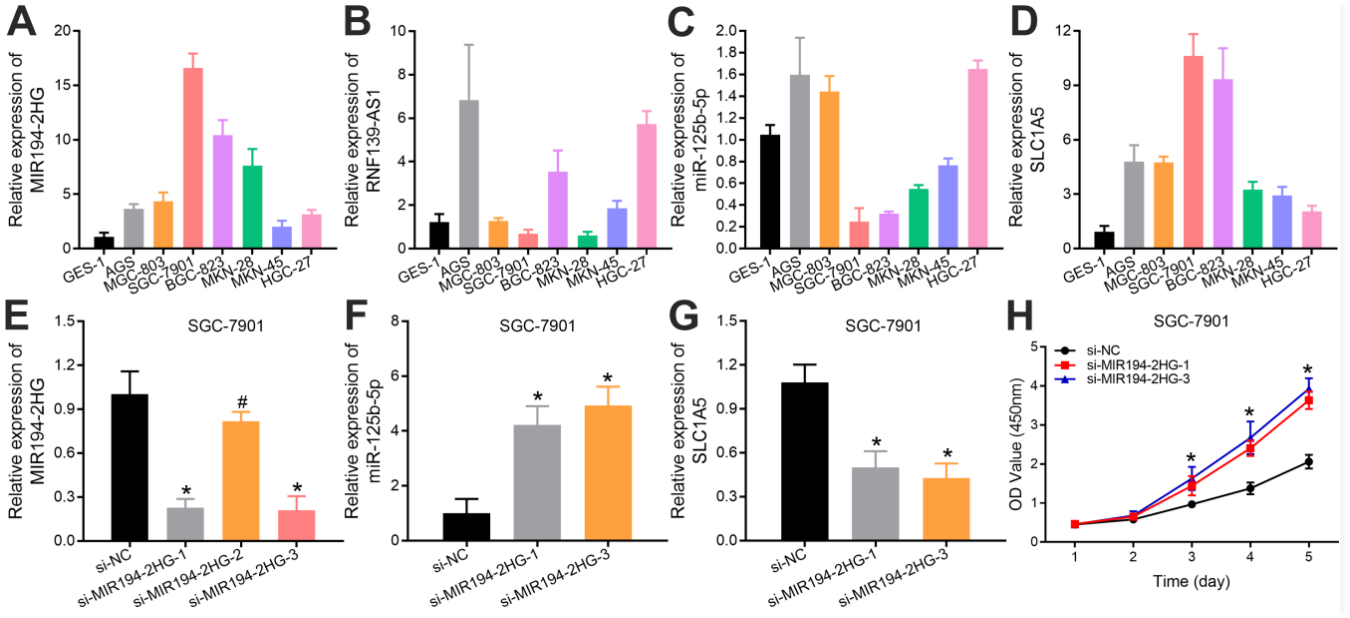
Relative expression levels of (**A**) MIR194-2HG, (**B**) RNF139-AS1, (**C**) hsa-miR-125b-5p and (**D**) SLC1A5 in 7 GC cell lines (AGS, MGC-803, SGC-7901, BGC-823, MKN-28, MKN-45, HGC-27) and 1 normal gastric mucosal epithelial cell line (GES-1). The (**E**) MIR194-2HG expression in SGC-7901 cells, which were transfected with si-MIR194-2HG and control vectors, were validated using quantitative real-time PCR. The effect of MIR194-2HG on the expression levels of (**F**) hsa-miR-125b-5p and (**G**) SLC1A5 was examined in SGC-7901 cells with MIR194-2HG knockdown. (**H**) MIR194-2HG silencing increased the activity of GC cells *in vitro* by CCK8 assays (*p-value <0.05; # p-value >0.05).

In order to verify the aforementioned results of bioinformatics analysis, we further detected the expression levels of MIR194-2HG, RNF139-AS1, hsa-miR-125b-5p and SLC1A5 in 1 normal gastric mucosa epithelial cell line (GES-1) and 7 GC cell lines (AGS, MGC-803, SGC-7901, BGC-823, MKN-28, MKN-45, HGC-27) by qRT-PCR, respectively. We found that the expression levels of MIR194-2HG and SLC1A5 in GC cells were significantly higher than those in normal gastric mucosa epithelial cells (Figure 4A, 4D). However, the expression levels of RNF139-AS1 and hsa-miR-125b-5p were inconsistent in different GC cell lines compared with normal gastric mucosa epithelial cells, as RNF139-AS1 showed higher expression in 5 GC cell lines and hsa-miR-125b-5p exhibited lower expression in 4 GC cell lines, which may be related to the selection of cell lines (Figure 4B, 4C). Therefore, we mainly examined the potential ceRNA network of MIR194-2HG, hsa-miR-125b-5p and SLC1A5. According to the different expression levels of MIR194-2HG in GC cells, we selected the SGC-7901 cell line, which had the highest MIR194-2HG expression for further study. We first transfected SGC-7901 cells with si-MIR194-2HG as well as their control groups, and evaluated their MIR194-2HG expression by qRT-PCR (Figure 4E). In addition, we evaluated the expression of hsa-miR-125b-5p and SLC1A5 in the SGC-7901 cells with MIR194-2HG knockdown. The results showed that, compared with the control groups, the expression of hsa-miR-125b-5p was up-regulated, while the expression of SLC1A5 was relatively decreased (Figure 4F, 4G). To some extent, we proved that MIR194-2HG regulates the expression of hsa-miR-125b-5p and SLC1A5 in GC cells. The knockout of MIR194-2HG in GC cells leads to the decrease of ceRNA binding to hsa-miR-125b-5p, which is the reason for the up-regulation of hsa-miR-125b-5p expression. The up-regulation of hsa-miR-125b-5p expression leads to the increase of ceRNA binding to SLC1A5 in GC cells, and a large number of ceRNA binding to SLC1A5 leads to the down-regulation of SLC1A5 expression. Furthermore, CCK-8 assays showed that knockdown of MIR194-2HG significantly increased GC cell proliferation, respectively, in a time-dependent manner, compared with the control groups *in vitro* (Figure 4H). All these proved that MIR194-2HG may serve as an inhibitory factor in the progression of GC.

### Validation of the results of bioinformatics analysis of TCGA by lnCAR database and GEPIA2 database

**Figure 5:**
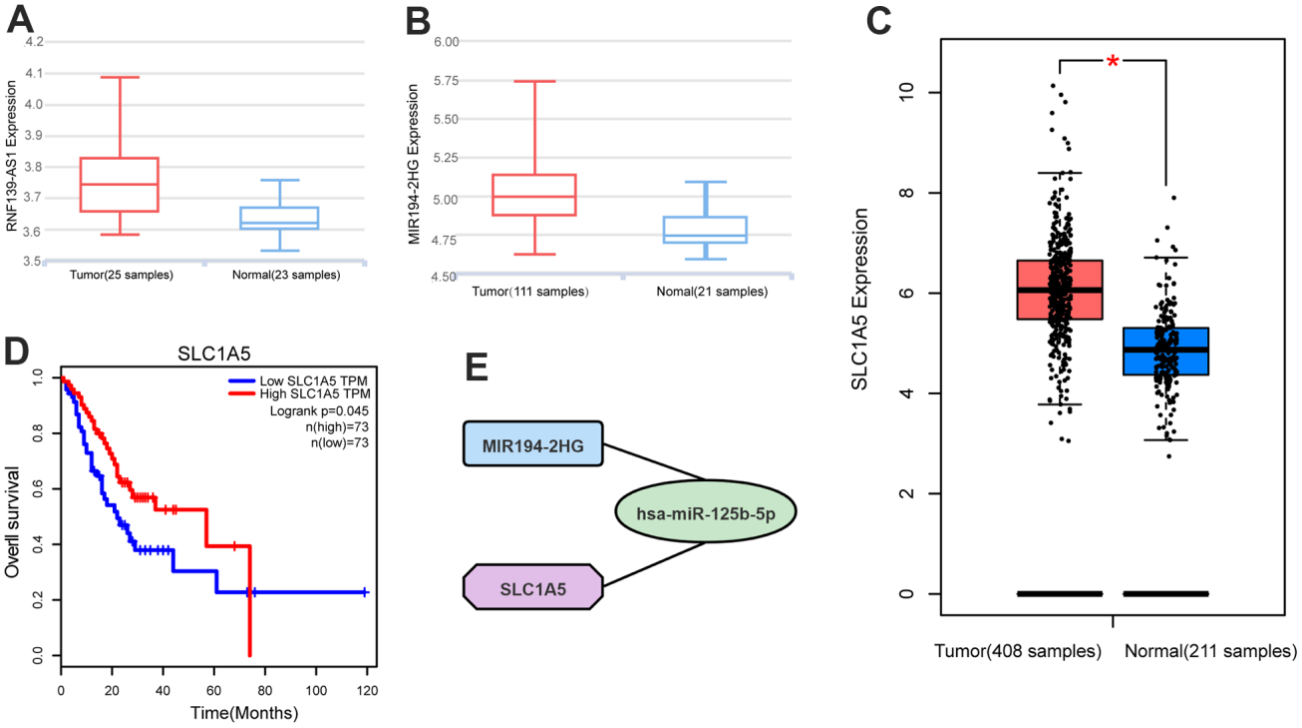
Differential expression analysis of (**A**) RNF139-AS1, (**B**) MIR194-2HG and (**C**) SLC1A5 in normal samples and GC samples by GTEx database, Ensembl database and Refgene database. Survival analysis by the GEPIA2 database shows (**D**) SLC1A5 is associated with overall survival. (**E**) Competing endogenous RNAs networks. Quadrilateral (lncRNA: MIR194-2HG), circular (miRNA: hsa-miR-125b-5p), octagonal (mRNA: SLC1A5).

lnCAR is a comprehensive database that is specifically dedicated to displaying differential expression profiles in human cancers by re-annotating microarray probes. The lncRNA data of lnCAR database comes from the Ensembl database (https://asia.ensembl.org/index.html) and the Refgene database (https://www.ncbi.nlm.nih.gov/refseq/). Through the lnCAR database (https://lncar.renlab.org/), we found that the expression of RNF139-AS1 and MIR194-2HG was relatively high in GC samples compared with normal samples (Figure 5A, 5B). We found that the expression of SLC1A5 in GC samples was relatively higher than that in normal samples in the GEPIA2 database (http://gepia.cancer-pku.cn/) (Figure 5C). Using the GEPIA2 database, we found that GC patients with high expression of SLC1A5 had a longer survival time than patients with low expression of GC (Figure 5D).

Through bioinformatics analysis and cell experiment verification, the ceRNA network of the ferroptosis-related gene (SLC1A5) was constructed. The simplified ceRNA network consists of one lncRNA, one miRNA and one ferroptosis-related mRNA, with a total of two nodes (Figure 5E).

### Results of the correlation analysis between SLC1A5 and immune infiltration, immune cell marker genes, immune checkpoint genes

**Figure 6:**
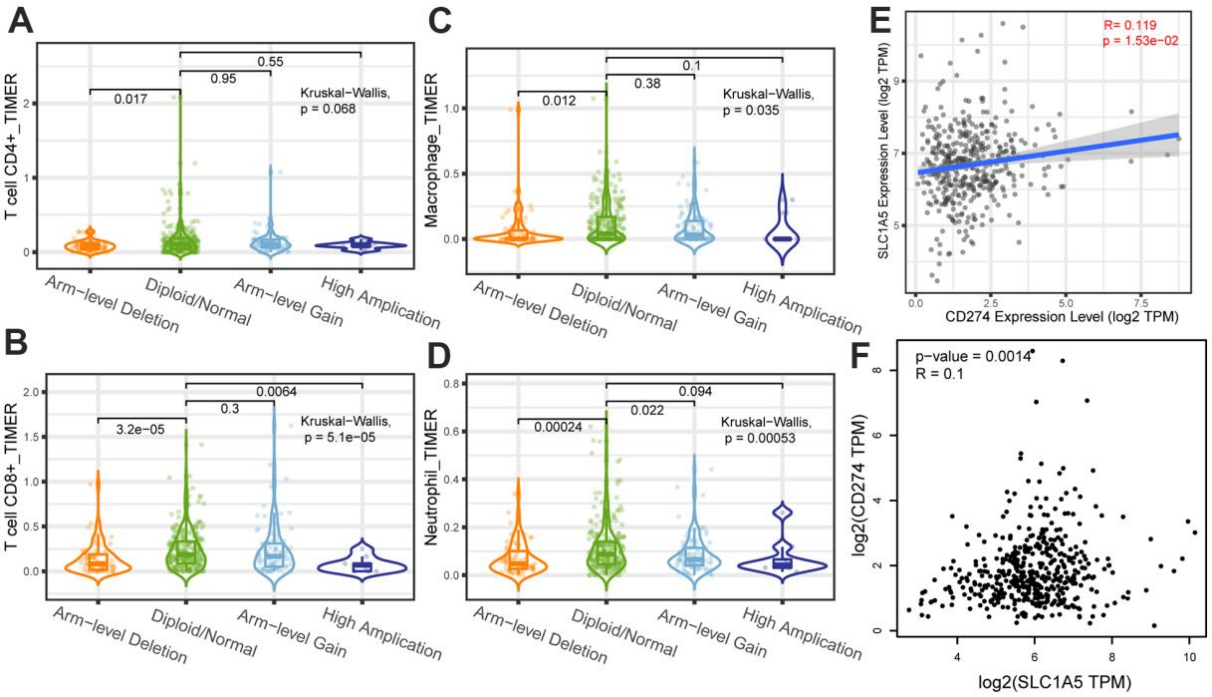
The correlation analysis between copy number variation of SLC1A5 and infiltrating immune cells (**A**) CD4+T cells, (**B**) CD8+T cells, (**C**) macrophages, (**D**) neutrophils. The x-axis depicts the copy number of SLC1A5 and the y-axis depicts the log-fold of immune infiltration levels. The correlation analysis between the expression level of SLC1A5 and the expression level of immune checkpoint gene (**E**, **F**) CD274.

**Figure 7:**
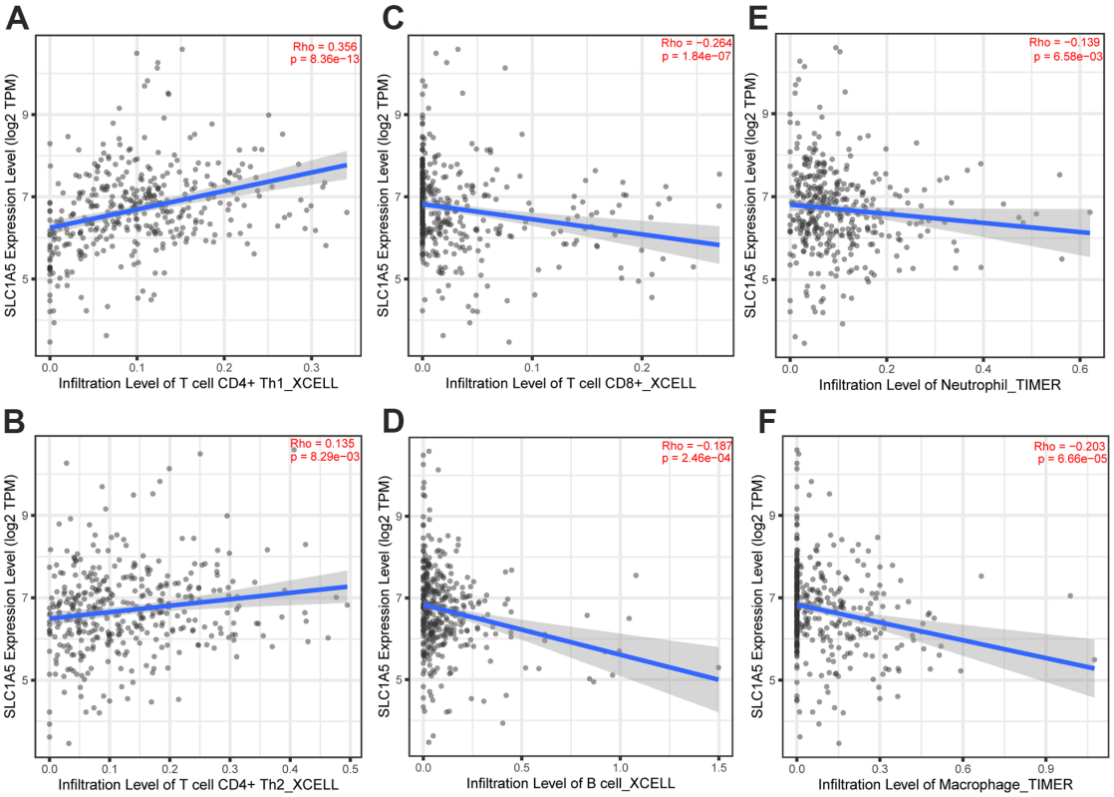
**The correlation analysis between the expression level of SLC1A5 and the infiltration level of immune cells.** The expression level of SLC1A5 was positively correlated to the infiltration level of (**A**, **B**) CD4+T cells. The expression of SLC1A5 was negatively correlated with the number of (**C**) CD8+T cells, (**D**) B cells, (**E**) neutrophils and (**F**) macrophages.

**Figure 8:**
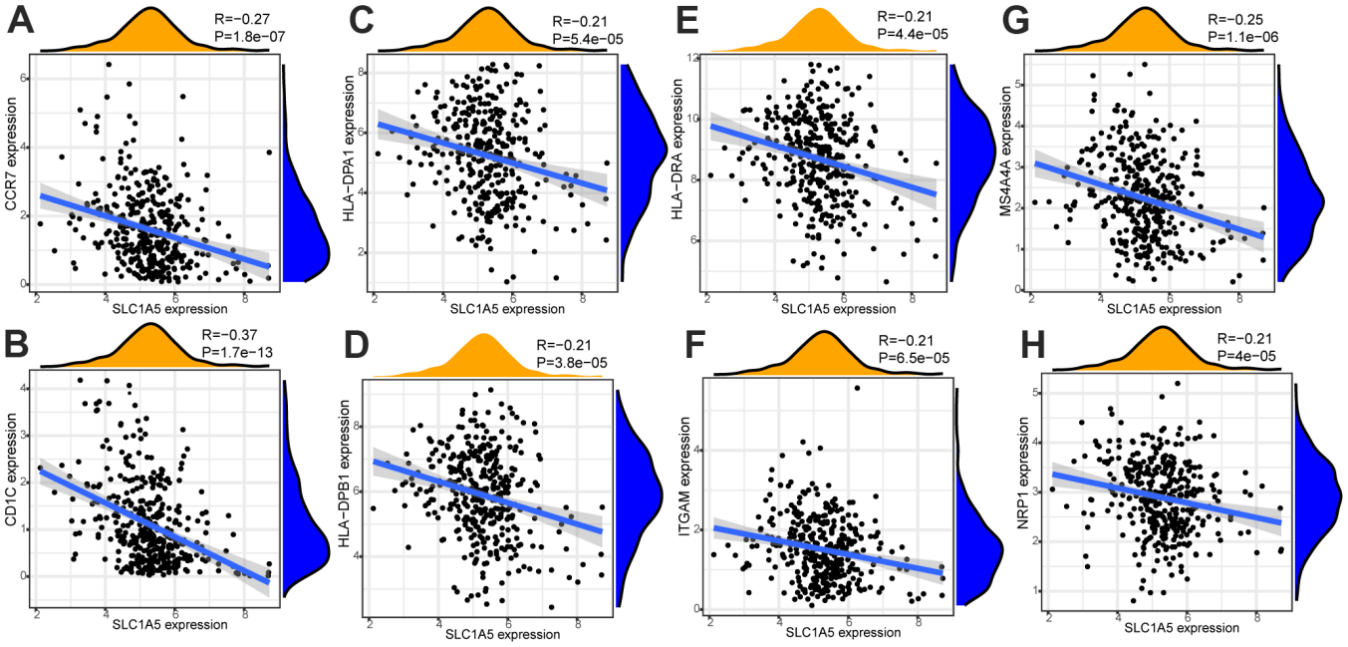
**The correlation analysis between the expression level of SLC1A5 and the expression level of immune cell marker genes.** The expression level of immune cell marker genes (CCR7 (**A**), CD1C (**B**), HLA-DPA1 (**C**), HLA-DPB1 (**D**), HLA-DRA (**E**), ITGAM (**F**), MS4A4A (**G**), NRP1(**H**)) were associated with the expression level of SLC1A5 (R<-0.02, p-value <0.001).

Through Tumor immune estimation resource (TIMER) analysis, The Arm-level deletion/gain and High Amplification of SLC1A5 can lead to changes in infiltration levels of immune cells. In GC, when the copy number of SLC1A5 changed, the number of infiltrating immune cells decreased (Figure 6A–6D). The expression correlation analysis between SLC1A5 and immune checkpoint genes showed that there was a positive correlation between the expression level of CD274 and SLC1A5 (Figure 6E, 6F). XCELL found that the expression level of SLC1A5 was related to the infiltration level of B cells, CD8+T cells and CD4+T cells. When the expression level of SLC1A5 increased, the number of infiltrating B cells and CD8+T-cells decreased, while the number of infiltrating CD4+Tcells was positively correlated with the expression level of SLC1A5. TIMER found that the expression of SLC1A5 was negatively correlated with the number of macrophages and neutrophils. As shown in Figure 7. The expression correlation analysis between SLC1A5 and immune cell marker genes showed that the expression level of 16 immune cell marker genes (CD19, CD79A, CD8A, CD4, CD163, VSIG4, MS4A4A, CEACAM8, ITGAM, CCR7, HLA-DPB1, HLA-DRA, HLA-DPA1, CD1C, NRP1, ITGAX) were associated with the expression level of SLC1A5. Among them, only one immune cell marker gene (CEACAM8) was positively correlated with the expression level of SLC1A5. The remaining 15 immune cell marker genes were negatively correlated with the expression level of SLC1A5 (Supplementary Table 5). The correlation scatter diagram is shown in Figure 8 (R<-0.02, p-value <0.001).

## DISCUSSION

In this study, the expression of lncRNAs, miRNAs and mRNAs were analyzed comprehensively using bioinformatics data, and a ceRNA network was formed. KM and Cox survival analysis showed that the ferroptosis-related gene (SLC1A5) was associated with the prognosis of GC, suggesting the potential role of SLC1A5 in GC. The occurrence and reaction of ferroptosis are due to the lipid peroxidation induced by Fe2+ and transferrin, which leads to the disorder of the GSH system and inhibition of its core regulatory enzyme GPX4. In cells, glutamate and cysteine eventually synthesize GSH to assist GPX4 in maintaining intracellular redox homeostasis. SLC1A5 is a cell surface transporter that mediates the uptake of neutral amino acids including cysteine and glutamine [7]. In addition, we carried out experimental verification of the results of bioinformatics analysis. The differential expression of MIR194-2HG and SLC1A5 in GC cells and the normal human gastric mucosa epithelial cells was confirmed by qRT-PCR. Through the cck8 experiment, we found that the survival rate of GC cells with MIR194-2HG knockout was significantly increased. This proves that MIR194-2HG may be an inhibitory factor in the progression of GC. In this study, MIR194-2HG and hsa-miR-125b-5p are all related to the survival time of patients with GC and can be used as potential biomarkers to judge the prognosis. Through high-throughput sequencing analysis of the transcriptome, the researchers found that MIR194-2HG was down-regulated in bladder cancer [8]. Other studies have found that hsa-miR-125b-5p has a regulatory effect on human osteoarthritis (OA) induced by interleukin-1 [9]. However, the role of MIR194-2HG and hsa-miR-125b-5p and their underlying mechanisms in cancers including GC are still largely unknown. In this study, hsa-miR-125b-5p plays an important role in the ceRNA network. The hsa-miR-125b-5p can bind to SLC1A5 and inhibit the expression of SLC1A5. The MIR194-2HG can bind to hsa-miR-125b-5p, thus relieving the inhibition of SLC1A5 expression by hsa-miR-125b-5p. We will explore the biological functions of SLC1A5, MIR194-2HG and hsa-miR-125b-5p in future research.

With the deepening of research, researchers have made great progress in understanding the role of ferroptosis-related genes in tumorigenesis and development. The researchers found that the engagement of ferroptosis in the activities of tumor suppressors, establishes a natural barrier to cancer development [10]. However, ferroptosis escape mediated by oncogenes or oncogene signals contributes to the initiation, progression and metastasis of tumors [11]. The unique metabolism of cancer cells, their high load of reactive oxygen species (ROS) and their specific mutations make some of them prone to ferroptosis, thus exposing the vulnerabilities that can be used as therapeutic targets in some cancer types [12, 13]. This provides a promising direction for the enrichment and development of cancer treatment. Recent studies have also shown that the tumor microenvironment (TME) determines whether tumor cell ferroptosis will occur. CD8+ cytotoxic T cells are the main executors of anti-tumor immunity in TME. They secrete interferon-γ, and then inhibit cystine uptake in tumor cells by down-regulating the expression of ferroptosis-related genes (SLC7A11), thus enhancing ferroptosis in tumors. It is suggested that ferroptosis plays an important role in T cell-mediated antitumor activity, and blocking ferroptosis-related gene-mediated amino acid uptake is a potential cancer treatment strategy [14, 15].

In recent years, the mechanism of ferroptosis on tumors has been a hot research field, but the potential mechanism of tumor immunity and ferroptosis-related genes is still little known, which is worthy of further study. Some previous studies have shown that SLC1A5 may cause ferroptosis of tumor cells, but the correlation between SLC1A5 and immune infiltration in GC is still largely unknown. Immune cell infiltration in the TME plays an important role in the occurrence and development of tumors [16, 17]. Tumor-infiltrating lymphocytes (TILs) are composed of CD8+T cells, CD4+T cells, regulatory T cells, macrophages, neutrophils, myeloid suppressor cells and natural killer cells, which interact with each other and play an anti-tumor or pro-tumor effect [18]. Lymphocyte infiltration in the TME is an independent predictor of the survival of cancer patients [19, 20]. Infiltrating immune cells may be used as tumor antagonistic factors or tumor-promoting factors [21, 22]. Cancer cells lead to the development of cancer by acquiring tumor antagonistic function that inhibits immune cells and the ability to evade immune surveillance [23]. The increased expression level of regulatory T cells, macrophages, neutrophils and myeloid suppressor cells usually indicates a poor prognosis [24]. Some studies have also shown that tumor-associated macrophages are associated with poor prognosis in patients with GC [25]. Tumor-infiltrating neutrophils can also inhibit normal T cell immunity and is related to the poor survival rate of GC [26]. Through the analysis of TIMER, it is found that The Arm-level deletion/gain and High Amplification of SLC1A5 can lead to changes in infiltration levels of immune cells. In GC, when the copy number of SLC1A5 changes, the number of infiltrating immune cells decreases. XCELL database analysis showed that the expression level of SLC1A5 in GC was negatively correlated with the infiltration level of B cells and CD8+T cells, positively correlated with the infiltration level of CD4+T cells. TIMER database analysis showed that the infiltration levels of macrophages and neutrophils were negatively correlated with the expression level of SLC1A5. These results suggest that low levels of SLC1A5 can enhance the activation of the immune system by increasing the infiltration of immune cells. SLC1A5 is a cell surface transporter responsible for the transport of neutral amino acids [27]. The function of SLC1A5 may be one of the factors affecting immune infiltration. The expression level of SLC1A5 was negatively correlated with the expression level of 15 immune cell marker genes (CD19, CD79A, CD8A, CD4, CD163, VSIG4, MS4A4A, ITGAM, CCR7, HLA-DPB1, HLA-DRA, HLA-DPA1, CD1C, NRP1, ITGAX), and only one immune cell marker gene (CEACAM8) was positively correlated with the expression level of SLC1A5. The expression correlation between SLC1A5 and immune cell marker genes suggested that SLC1A5 can regulate tumor immunity of GC through a variety of immune cell populations. In recent years, immunotherapy for immune microenvironment regulation and immune checkpoint regulation has shown certain efficacy in cancer treatment [28, 29]. The expression correlation analysis between SLC1A5 and immune checkpoint genes showed that there was a positive correlation between the expression level of the immune checkpoint gene (CD274) and SLC1A5, which suggested that we can further explore new treatments for GC through the study of SLC1A5. Therefore, through the correlation analysis of immune, we have reason to think that the ferroptosis-related gene SLC1A5 is closely related to tumor immunity, and the abnormal expression of SLC1A5 may change the tumor immune microenvironment, and the impairment of anti-tumor immunity may also be the reason for the progression and poor prognosis of GC.

To sum up, we speculate that these ceRNAs may be involved in the occurrence and development of GC, which have a good clinical research value. Our research found that MIR194-2HG, hsa-miR-125b-5p and SLC1A5 may be the potential diagnosis, prognosis biomarkers and treatment targets of GC. It was also found that SLC1A5 was closely related to the immune infiltration of GC. Through bioinformatics analysis and cell experiment verification, the ceRNA network of the ferroptosis-related gene (SLC1A5) was constructed and the relationship between the ferroptosis-related gene (SLC1A5) and MIR194-2HG, hsa-miR-125b-5p were systematically analyzed. MIR194-2HG affects the related function of SLC1A5 through the binding of hsa-miR-125b-5p. The new method for studying the function and mechanism of ferroptosis-related ceRNAs in human tumors by using publicly available gene data was shared.

## MATERIALS AND METHODS

### Data collection

Download the expression data and clinical survival data of TCGA about stomach adenocarcinoma (STAD) from UCSC Xena (https://xenabrowser.net/). The expression data included transcriptome data and lncRNA data. There are 407 samples, including 32 normal samples and 375 GC samples. The clinical survival data include the survival time and survival status of patients with GC, with a total of 502 samples, including 88 normal samples and 414 GC samples. We downloaded miRNA data from TCGA (https://portal.gdc.cancer.gov). According to miRNA data, there are 491 samples, including 45 normal samples and 446 GC samples. And the level 3 data was analyzed. Then, 60 ferroptosis-related genes were obtained by searching related literature [30] and are provided in Supplementary Table 6.

### The ferroptosis-related genes needed for the research were obtained by screening

The differentially expressed genes between GC samples and normal samples in the TCGA cohort were screened by R language version 4.1.1 LIMMA software package. The FC was used for screening, and the screening criteria for differential expression were | log_2_FC | > 1 and FDR < 0.05. The OS was analyzed by Kaplan-Meier analysis and Cox analysis using R language version 4.1.1 survival software package (p < 0.05). The ferroptosis-related genes with prognostic values were screened. Finally, the required gene: SLC1A5 was obtained by screening.

### The miRNAs with the targeted relationship with mRNAs were obtained by screening

Filter out the miRNAs combined with SLC1A5 from the Starbase database (http://starbase.sysu.edu.cn/). The Starbase database is mainly focused on miRNA-target interactions. It identifies more than 4.1 million miRNA-ncRNA, 2.9 million miRNA-mRNA, 4.1 million RBP-RNA and 1.5 million RNA-RNA interactions from multi-dimensional sequencing data. It is an open-source platform for studying the miRNA-ncRNA, miRNA-mRNA, ncRNA-RNA, RNA-RNA, RBP-ncRNA and RBP-mRNA interactions from CLIP-seq, degradome-seq and RNA-RNA interactome data. The miRNA-mRNA expression correlation analysis was carried out by using R language version 4.1.1, and the miRNAs with significantly negative correlation with SLC1A5 were screened out. The screening criteria were spearman COR < -0.2 and p-value < 0.01. The differentially expressed miRNAs were screened from the miRNAs negatively related to SLC1A5 by using the R language version 4.1.1 LIMMA software package. Because the expression of SLC1A5 is up-regulated in GC samples, to obtain miRNA with a negative correlation with SLC1A5, we set the screening criteria for differential expression genes to log2FC < 0andp-value < 0.05. The expression of the screening criteria for differential expression genes is up-regulated in gastric cancer samples. Then, the survival software package of R language version 4.1.1 was used for survival analysis to screen the miRNAs with prognostic values. Finally, the miRNA:hsa-miR-125b-5p that meets the requirements was obtained by screening.

### The lncRNAs with the targeted relationship with miRNAs were obtained by screening

Filter out the lncRNAs combined with hsa-miR-125b-5p from the Starbase database. The miRNA-lncRNA expression correlation analysis was carried out by using R language version 4.1.1, and the lncRNAs with significantly negative correlation with hsa-miR-125b-5p were screened out. The screening criteria were spearman COR < -0.2 and p-value < 0.01. The differentially expressed lncRNAs were screened from the lncRNAs negatively related to hsa-miR-125b-5p by using the R language version 4.1.1 LIMMA software package. The screening criteria for differential expression genes were log_2_FC > 0 and p-value < 0.05. Finally, the R language version 4.1.1 survival software package was used for survival analysis to screen the lncRNAs (RNF139-AS1 and MIR194-2HG) with prognostic values.

### Cell culture and qRT-PCR validation

The GC cell lines (AGS, MGC-803, SGC-7901, BGC-823, MKN-28, MKN-45, HGC-27) and the normal gastric mucosa epithelial cell line (GES-1) were maintained in our lab. The cells were cultured in RPMI 1640 Medium (Biological Industries, Kibbutz Beit-Haemek, Israel) supplemented with 10% fetal bovine serum (Excell Bio, Shanghai, China) in a humidified environment at 37° C and 5% CO2. Total RNA was extracted from cells using the RNA Easy Fast Cell Kit from Tiangen Biotech Co., Ltd. (Tiangen, Beijing, China) according to the manufacturer’s instructions. Reverse transcription was carried out using the FastKing Reverse Transcription Kit (Tiangen) according to the manufacturer’s instructions. The cDNA synthesis of hsa-miR-125b-5p was carried out according to the manufacturer’s instruction with the TaqMan microRNA reverse transcription kit (Tiangen). The qRT-PCR was performed using the qPCR Kit (Tiangen) on the QuantStudio™ 5 System (Applied Biosystems by Thermo Fisher Scientific, Foster City, CA, USA). The 2−ΔΔCt was used to calculate the gene expression level. The qRT-PCR reaction was repeated 3 times. Primers for qRT-PCR were synthesized by Tsingke Biotechnology Co., Ltd. (Tsingke, Beijing, China). The concrete sequences of different primers were used as follows: RNF139-AS1, forward: 5′-CCAACAGGAACCATGAGC -3′, reverse: 5′-GGATTATGACGCAGTGTGG-3′; MIR194-2HG forward: 5′-GCCCTGTGTCCCCAAGT-3′, reverse: 5′-GCTGTCTCTCTGCCCCAT-3′; SLC1A5 forward: 5′-GGGAGGCTTTCTCTGGCT-3′, reverse: 5′-ACACTGAGGGCTGGGATG-3′. hsa-miR-125b-5p: GTCGTATCCAGTGCAGGGTCCGAGGTATTCGCACTGGATACGACTCACAA, hsa-miR-125b-5p-rtF: CGCGTCCCTGAGACCCTAAC, hsa-miR-125b-5p-rtR: AGTGCAGGGTCCGAGGTATT.

### Knock out MIR194-2HG in SGC-7901 cells by siRNA

In order to inhibit the expression of MIR194-2HG in GC cells, the specific small interfering RNA (siRNA) against MIR194-2HG (si-MIR194-2HG) was purchased from Jima Gene Technology Co., Ltd. (Jima, Shanghai, China). Following culturing the cells in 24-well plates at 37° C for 24 hours, the si-MIR194-2HG and the negative control (si-NC) were transfected into the GC cells (2x105 cells/well) using Lipofectamine 3000 according to the method of the manufacturer. In order to find the GC cells with the best knockout efficiency, three different siRNAs were used to transfect the SGC-7901 cells. After transfection for more than 24 hours, the MIR194-2HG-silenced GC cells were used for further study. Then the expression of the related RNA in GC cells with MIR194-2HG knockout was determined by qRT-PCR. The sequences used were as follows: si-MIR194-2HG-Homo-1515 forward, 5'-CUGCCCAGGAGUUGUAAAUTT-3' and reverse, 5'-AUUUACAACUCCUGGGCAGTT-3'. si-MIR194-2HG-Homo-2427 forward, 5'-GGUGCUGGUUUCUGCUUAUTT-3' and reverse, 5'-AUAAGCAGAAACCAGCACCTT-3'. si-MIR194-2HG-Homo-771 forward, 5'-CGGCAGGUUUGUGUAUCUATT-3' and reverse, 5'-UAGAUACACAAACCUGCCGTT.

### CCK-8 assay

The GC cells transfected with si-MIR194-2HG were inoculated into 96-well plates at 37° C for 48 hours (2x105 cells/well). CCK8 reagent (10 μl) was added into each well, and the mixture was incubated with the cells for 2h. The absorbance of each well was measured at the wavelength of 450 nm using a multi-function microplate reader at 1-5 days post-cell culture.

### The correlation between immune infiltration, immune cell marker genes, immune checkpoint genes and SLC1A5 was analyzed

The correlation analysis between copy number variation (CNV) of SLC1A5 and the immune infiltration was carried out by TIMER (http://timer.comp-genomics.org). The correlation analysis between the expression level of SLC1A5 and the immune infiltration was carried out by TIMER and the Cell types enrichment analysis was carried out by XCELL (https://xcell.ucsf.edu/). The correlation analysis between the expression level of SLC1A5 and the expression level of immune cell marker genes was carried out by R language version 4.1.1. The correlation analysis between the expression level of SLC1A5 and the expression level of immune checkpoint genes was carried out by TIMER and GEPIA (http://gepia.cancer-pku.cn/).

## Supplementary Material

Supplementary Tables
