# Supplementary material for: Identification of the ferroptosis-related ceRNA network related to prognosis and tumor immunity for gastric cancer
Source: Aging (Albany NY). 2022 Jul 14;14(14):5768–82. doi: 10.18632/aging.204176 (PMC9365562; doi:10.18632/aging.204176)
Supplement: Supplementary Tables [file aging-14-204176-s001.pdf]

SUPPLEMENTARY TABLES

Supplementary Table 1. Eight differentially expressed ferroptosis-related genes.

Gene	conMean	treatMean	logFC	P-Value	FDR
FANCD2	0.719778188	1.779459893	1.059681705	0.000000000000000214	0.0000000000000112
AKR1C2	3.121942579	1.142276251	-1.979666328	0.000000000000321	0.00000000000834
AKR1C1	3.35703264	1.526822073	-1.830210567	0.000000000000979	0.000000000017
NOX1	0.233717386	1.331700366	1.09798298	0.00000000000305	0.0000000000397
SLC1A5	3.911192645	5.259044624	1.347851979	0.0000000000045	0.0000000000468
TFRC	3.355113781	4.650328414	1.295214633	0.0000000000444	0.00000000033
CRYAB	4.576554607	2.410610525	-2.165944082	0.0000000000781	0.000000000508
MT1G	5.926700692	4.46376449	-1.462936202	0.000301396	0.000681418

Supplementary Table 2. Survival analysis of SLC1A5 by KM and COX method.

Gene	HR	HR.95L	HR.95H	Cox P-Value	KM P-Value
SLC1A5	0.826930504	0.704241686	0.971	0.0204	0.031562055

Supplementary Table 3. According to the optimal cutpoint, gastric cancer samples were divided into two groups with high and low expression, and survival analysis was performed for hsa-miR-125b-5p, hsa-miR-199b-5p, RNF139-AS1 and MIR194-2HG.

miRNA/lncRNA	Cutpoint	P-Value
hsa-miR-125b-5p	8.341448549	0.000947993
hsa-miR-199b-5p	6.172403905	0.007452684
RNF139-AS1	0.334214866	0.032830804
MIR194-2HG	1.172825	0.035368436

Supplementary Table 4. Expression correlation analysis between SLC1A5 and hsa-miR-125b-5p, hsa-miR-199b-5p.

miRNA/lncRNA	Gene/miRNA	Cor	P-Value	logFC	Diff Pvale
hsa-miR-199b-5p	SLC1A5	-0.229630574	0.00000813	0.52	0.000545
hsa-miR-125b-5p	SLC1A5	-0.220281113	0.000019	-0.721	0.0000895
RNF139-AS1	hsa-miR-125b-5p	-0.248230192	0.00000135	0.242	0.00000000000669
MIR194-2HG	hsa-miR-125b-5p	-0.261551566	0.000000343	0.565	0.000172

Differential expression analysis of hsa-miR-125b-5p and hsa-miR-199b-5p in normal samples and gastric cancer samples. Expression correlation analysis between hsa-miR-125b-5p and RNF139-AS1, MIR194-2HG. Differential expression analysis of RNF139-AS1 and MIR194-2HG in normal samples and gastric cancer samples.

Supplementary Table 5. The expression correlation analysis between SLC1A5 and 16 immune cell marker genes (CD19, CD79A, CD8A, CD4, CD163, VSIG4, MS4A4A, CEACAM8, ITGAM, CCR7, HLA-DPB1, HLA-DRA, HLA-DPA1, CD1C, NRP1, ITGAX).

Immune cell	Gene	Cor	P-Value
B cell	CD19	-0.1900512	0.00021947
B cell	CD79A	-0.196561611	0.000131321
CD8+ T cell	CD8A	-0.157800432	0.002201165
CD4+ T cell	CD4	-0.179703721	0.000480036
M2 macrophage	CD163	-0.166586415	0.001221057
M2 macrophage	VSIG4	-0.167709865	0.001130085
M2 macrophage	MS4A4A	-0.24903493	0.00000113
Neutrophil	CEACAM8	0.130505902	0.011418407
Neutrophil	ITGAM	-0.205231767	0.0000646
Neutrophil	CCR7	-0.266529298	0.00000018
Dendritic cell	HLA-DPB1	-0.211410399	0.0000383
Dendritic cell	HLA-DRA	-0.20983889	0.0000438
Dendritic cell	HLA-DPA1	-0.207275003	0.0000544
Dendritic cell	CD1C	-0.370220275	0.000000000000169
Dendritic cell	NRP1	-0.210939584	0.0000398
Dendritic cell	ITGAX	-0.181653658	0.000415511

Supplementary Table 6. 60 confirmed ferroptosis-related genes related to humans.

GSS	SAT1	RPL8	SQLE	PTGS2	MT1G	HSPB1	ALOX15	STEAP3	HMOX1
CS	TFRC	DPP4	ZEB1	FDFT1	AIFM2	PHKG2	AKR1C3	CHAC1	HMGCR
CBS	NFS1	GLS2	EMC2	PEBP1	ACSL4	ACACA	ALOX12	LPCAT3	SLC7A11
NQO1	NOX1	ACO1	GOT1	CISD1	G6PD	ABCC1	AKR1C2	FADS2	AKR1C1
RPL8	TP53	FTH1	KEAP1	HSBP1	ALOX5	GCLM	NCOA4	CARS1	ATP5MC3
CD44	GPX4	GCLC	PGD	IREB2	ACSF2	CRYAB	NFE2L2	SLC1A5	FANCD2
